# G6PD Deficiency Does Not Enhance Susceptibility for Acquiring *Helicobacter pylori* Infection in Sardinian Patients

**DOI:** 10.1371/journal.pone.0160032

**Published:** 2016-07-28

**Authors:** Maria Pina Dore, Giuseppina Marras, Chiara Rocchi, Sara Soro, Giovanni Mario Pes

**Affiliations:** 1 Dipartimento di Medicina Clinica e Sperimentale, University of Sassari, Sassari, Italy; 2 Baylor College of Medicine, Houston, Texas, United States of America; 3 Istituto Nazionale Biostrutture e Biosistemi, Sassari, Italy; Indian Institute of Science, INDIA

## Abstract

**Background:**

Subjects with glucose-6-phosphate dehydrogenase (G6PD) deficiency may be more susceptible to infections due to impaired leukocyte bactericidal activity. The disorder is common in the Mediterranean area. The aim of this study was to investigate whether G6PD deficiency may be a risk factor for acquiring *H*. *pylori* infection.

**Methods:**

We performed a retrospective study. Data from clinical records of 6565 patients (2278 men and 4287 women, median age 51, range 7‒94) who underwent upper endoscopy between 2002 and 2014 were collected. *H*. *pylori* status, assessed by histology plus rapid urease test or ^13^C-urea breath test, and G6PD status were also reported. A multiple logistic regression model was used to investigate the association between G6PD deficiency and *H*. *pylori* infection.

**Results:**

Enzyme deficiency was detected in 12% (789/6565) of the entire cohort, and more specifically in 8.3% of men and in 14.0% of women. Overall, the proportion of patients positive for *H*. *pylori* was 50.6% and 51.5% among G6PD deficient and non-deficient patients (χ² = 0.271; p = 0.315). Moreover, among G6PD-deficient and normal patients the frequency of previous *H*. *pylori* infection was similar. After adjustment for age and gender the risk for acquiring *H*. *pylori* infection was similar in G6PD-deficient and normal patients. Only age was a strong statistically significant risk predictor.

**Conclusions:**

These results demonstrate for the first time that G6PD deficiency does not enhance patients’ susceptibility to acquire *H*. *pylori* infection in Sardinia.

## Introduction

*Helicobacter pylori* infection affects more than 50% of the world population and is associated with various disorders of the proximal gastro‒intestinal tract such as chronic gastritis and duodenal ulcers, gastric adenocarcinoma and gastric lymphoma [1]. The infection is usually acquired in childhood [2, 3], and risk factors are related to socio˗economic status and living conditions [2–5]. Gastric colonization by the microorganism is also influenced by host defences including reactivity of immune cells [6], and efficiency of mechanisms against oxidative challenge [7, 8]. Although some of these conditions have been investigated in detail, a number of them remained largely unexplored.

D-glucose-6-phosphate dehydrogenase (G6PD, D-glucose-6-phosphate: NADP oxydoreductase; EC 1.1.1.49) is a cytoplasmic enzyme able to catalyze the first step of the pentose phosphate pathway, which plays a key role in producing the extra‒mitochondrial coenzyme nicotinamide-adenine dinucleotide phosphate (NADPH) as well as the ribose necessary to synthesize DNA [9]. G6PD deficiency is the most common inherited enzymatic disorder of red blood cells in humans. The G6PD gene maps on the long arm of X chromosome (Xq28 locus), hence deficiency is transmitted by a X-linked pattern [10]. Therefore, total enzyme deficiency is observed in hemizygote men and homozygote women, whereas variable levels of enzyme activity are detected in heterozygote women, resulting from partial compensation by the normal allele and random inactivation of one X chromosome [11]. Carriers of the G6PD deficient allele appear to have a selective advantage against malaria caused by *Plasmodium falciparum* [12]. For this reason, in areas of the world where malaria is endemic, the frequency of G6PD deficiency could be considerably increased.

Among hundreds of mutant alleles associated with G6PD deficiency, the *Mediterranean* mutation (C→T transition at nucleotide 563 of G6PD gene) has a frequency of about 12–24% in Sardinia, the highest in Italy [13, 14].

The majority of G6PD‒deficient individuals are entirely asymptomatic. However, subjects carrying the most common G6PD variants are subjected to recurrent acute hemolytic anemia. Hemolysis is triggered by infections, ingestion of *Vicia faba* (favism), and treatment with several pro‒oxidant drugs.

Deficient enzyme activity may impair protection from oxidative injury in cells other than erythrocytes [15]. More specifically, leukocytes from G6PD deficient subjects show a reduced response to bacterial challenge [16–21]. G6PD deficient granulocytes in G6PD *Mediterranean* variant displayed reduced function by 25% to 33% in most studies [22, 23], suggesting an increased susceptibility to bacterial infection. However, population studies on the incidence and prevalence of bacterial infections, and more specifically on *H*. *pylori* infection in G6PD deficiency patients are lacking.

Therefore, in this study we investigate a possible association between *H*. *pylori* infection and G6PD deficiency in a large patient cohort from Northern Sardinia.

## Materials and Methods

### Study population

Clinical records of patients scheduled for upper endoscopy at the Department of Internal Medicine, University of Sassari, Italy, from January 2002 to December 2014 were collected. Demographic data including gender and age were available.

In the case of multiple esophago-gastro-duodenoscopies (EGDs) for the same patient within the given time period, only results from the first procedure were included in the analysis.

#### Inclusion criteria

Only patients with a known *H*. *pylori* status were included in the study. The infection was assessed according to histological findings on the gastric biopsies plus a positive ^13^C urea breath test (^13^C-UBT) or rapid urease testing. A minimum of four gastric biopsy specimens were available for each patient: two from the *antrum*, one from the *angulus*, and one from the *corpus* of the stomach. The presence of *H*. *pylori* and active ‒ chronic gastritis, follicular gastritis, intestinal metaplasia, atrophy and dysplasia was assessed by an expert Gastro-Intestinal pathologist, based on tissue morphology [5]. Since the purpose of this study was to assess the susceptibility to *H*. *pylori* infection in G6PD deficient subjects—regardless of the type of gastritis—active gastritis, intestinal metaplasia, follicular gastritis and atrophy were reported as present if they were observed in at least one biopsy irrespective of the site [5]. *H*. *pylori* infection and active gastritis associated with intestinal metaplasia and/or atrophy was considered to be a consequence of *H*. *pylori* infection. The presence of intestinal metaplasia and/or atrophy in the gastric biopsies without *H*. *pylori* infection was considered evidence of a past infection.

### Ethical Considerations

An Institutional Review Board approval was obtained from *Comitato di Bioetica*, *Azienda Ospedaliero-Universitaria di Sassari* (Prot N° 2099/CE, 2014). Since only pre-existing charts were used, all patient records were de-identified before the analysis and there was no need to gather informed consent from participants.

### G6PD assay

G6PD activity was determined in all patients with a quantitative assay based on the ratio between G6PD/6GPD in erythrocytes, by using a standard routine enzymatic assay. Genotyping analysis was not performed in G6PD deficient patients.

### Statistical analysis

All patients were stratified by age in 10-year intervals. The distribution of patients according to gender and age (at the time of EGD) was calculated and expressed as a percentage. Based on histological features, patients were classified as having: i) Active *H*. *pylori* infection (chronic active gastritis positive for *H*. *pylori*); ii) Past *H*. *pylori* infection (metaplasia and/or atrophy negative for *H*. *pylori* infection); and iii) Long-standing *H*. *pylori* infection (metaplasia and/or atrophy positive for *H*. *pylori* infection). Results of G6PD activity were recorded as a dichotomous variable (deficient/normal) and mild or moderate G6PD activity was considered normal for the analysis.

The association between G6PD and *H*. *pylori* infection was expressed as the unadjusted odds ratios (ORs) and their 95% confidence intervals (CIs). The statistical power was calculated using the online DSS Research Statistical Power Calculator [24], on the basis of an alpha error level of 5%, while testing a 5% difference in frequency between G6PD deficient and non deficient subjects. Moreover, a multiple logistic regression model was fitted while controlling for potential confounding variables. For each covariate, the regression coefficients and their standard error were calculated as well as the ORs and their 95% CIs using the Wald formula (95%CI = OR^1±ß/SE^). Adjusted R² statistic was used to assess model fit. All statistical analyses were performed using SPSS statistical software (version 16.0, Chicago, IL, USA) p‒values <0.05 were considered statistically significant.

## Results and Discussion

**Fig 1:**
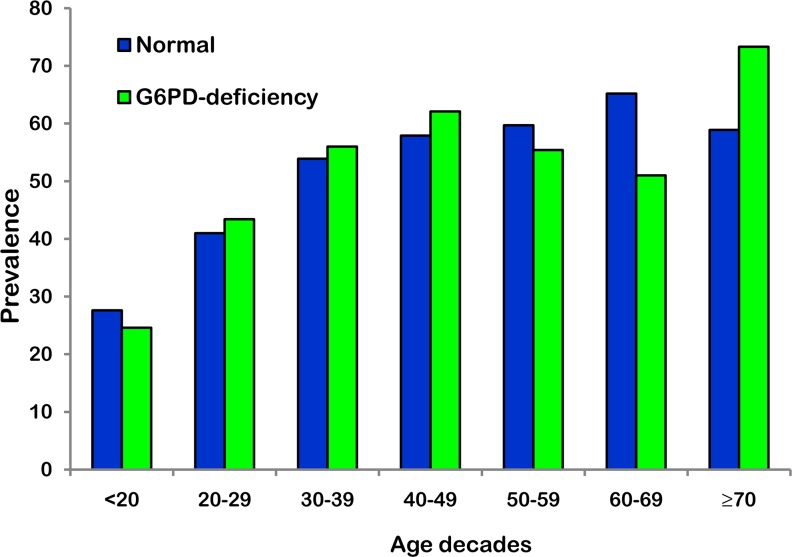
Prevalence of *H*. *pylori* infection according to age decade and D-glucose-6-phosphate dehydrogenase status.

A total of 6565 clinical records were available for the analysis (Table 1). The proportion of women was 65.3% (4287). Mean age was comparable between G6PD-deficient patients and controls (50.4 ± 16.9 vs 50.6 ± 17.3, p = 0.789). The entire study cohort was white Caucasian. Prevalence of *H*. *pylori* infection according to age decade and G6PD status is shown in Fig 1. Enzyme deficiency was recorded in 12% (789/6565) of the entire cohort, and more specifically in 8.3% of men and in 14.0% of women. Overall, the proportion of patients positive for *H*. *pylori* (active, past and long standing infection) was 50.6% and of 51.5% among G6PD deficient and non-deficient patients respectively, with no statistical difference (χ² = 0.271; p = 0.315). The statistical power given by our sample size was 84%, large enough to avoid the rejection of a false zero hypothesis of a 5% difference around a frequency of *H*. *pylori* of about 50%. Table 2 displays the unadjusted ORs and their 95% CIs for acquiring *H*. *pylori* infection among patients with and without G6PD deficiency.

**Table 1:** Demographic features of study population according to D-glucose-6-phosphate dehydrogenase status.

Variables	G6PD deficiency	G6PD normal
Patients No.	789	5776
Gender (M/F)	189/600	2089/3687
Mean age (years), men	52.8 ± 16.7	51.9 ± 17.2
Mean age (years), women	49.7 ± 16.9	49.9 ± 17.4
*H*. *pylori* infection	399/789 (50.6%)	2978/5776 (51.6%)

**Table 2:** Unadjusted odds ratio and 95% confidence interval for *H*. *pylori* infection among patients with and without G6PD deficiency undergoing upper endoscopy.

Variable	Total	Normal G6PD deficiency No. (%)	G6PD deficiency No. (%)	OR (95% CI)
H. pylori status				
No infection^‡^	3188	2798 (48.4)	390 (49.4)	1.00 Reference
Active infection^a^	2285	2018 (34.9)	267 (33.8)	0.95 (0.80–1.12)
Past infection^b^	898	794 (13.7)	104 (13.2)	0.94 (0.75–1.18)
Long standing^c^	194	166 (2.9)	28 (3.5)	1.21 (0.80–1.83)
Total patients	6565	5772	789	

^‡^Reference group

^a^Active *H*. *pylori* infection: chronic active gastritis with *H*. *pylori* infection

^b^Past *H*. *pylori* infection: metaplasia and/or atrophy without *H*. *pylori* infection

^c^Long-standing *H*. *pylori* infection: metaplasia and/or atrophy without *H*. *pylori* infection

Interestingly, the G6PD deficiency seems to entail a risk for active or past *H*. *pylori* infection similar to patients without enzyme deficiency. Patients with G6PD deficiency had a risk of long‒standing infection slightly increased, (OR = 1.21 [95% CI 0.80‒1.83]) although not significantly. Multiple logistic regression analysis is reported in Table 3.

**Table 3:** Multiple logistic regression analysis for G6PD status and age and gender associated with *H*. *pylori* infection.

Covariates	ORs	95% CI of OR	p-value
Age (years)			
<50	1.000	‒	‒
≥50	2.090	1.894‒2.307	<0.0001
Gender			
Women	1.000	‒	‒
Men	0.896	0.807‒0.995	0.039
G6PD			
Normal	1.000	‒	‒
Deficiency	0.966	0.830‒1.124	0.652

After adjustment for all covariates G6PD deficiency was not associated with a significantly increased risk for acquisition of *H*. *pylori* infection (OR = 1.02, 95% CI 0.827‒1.252). This lack of association was further confirmed also in the subgroups of patients with past infection (G6PD deficient 104/494; 21.1% vs G6PD normal 794/3598; 22.1%) (p = 0.643) suggesting that the clearance of infection was not affected by the G6PD status. Similarly, no difference was detected in subgroups of patients with long‒standing infection according to the G6PD status (G6PD deficient 28/418; 6.7% vs G6PD normal 166/2964; 5.6%) (p = 0.368). In the regression model the only strong significant predictor of *H*. *pylori* infection was age (OR = 1.81, 95% CI 1.572‒2.090).

Several loss‒of‒function mutations in the G6PD gene resulting in decreased erythrocyte enzymatic activity have been described. The distribution of the G6PD *Mediterranean* mutation in Sardinia varies according to historical and geographical pattern of malaria and altitude [25, 26] and the allele occurs in about 12% of the general population [13].

Since G6PD is a housekeeping enzyme, a number of studies focused on its role in different blood cells and tissues [27–29]. Cultured neutrophils, fibroblasts and lymphocytes displayed low levels of enzyme activity (8–15% of normal) and a marked reduction in the NADPH/NADP^+^ ratio [28, 29]. Several studies indicated that G6PD deficiency in leukocytes can result in chronic granulomatous disease with impaired host defense mechanisms against bacterial or fungal infection [30, 31]. For example G6PD-deficient epithelial cells in vitro showed a reduced tolerance to *Staphylococcus aureus* [18].

In one of the few studies in humans, Clark et al. reported a higher frequency of G6PD deficiency in patients hospitalized for infection in Iran significantly higher compared to non-infected control groups (p < 0.05) [32].

Previous studies reported a high prevalence of *H*. *pylori* infection in Sardinia [3, 33], making the island the ideal scenario to investigate the association between G6PD deficiency and *H*. *pylori* infection.

However, our study failed to detect a significant difference in the risk for acquiring *H*. *pylori* infection across subgroups of patients with normal or deficient G6PD enzyme activity. More specifically, no difference was observed among patients who acquired *H*. *pylori* infection at any time in their life (i.e. no matter if still active or past infection) and patients who never had contact with the pathogen, as demonstrated by the biopsy samples negative for the bacteria. The logistic regression model clearly showed that the major predictor of *H*. *pylori* infection was age, confirming the birth cohort effect [3]. The lack of association persisted after adjustment for age, and gender. Our results are consistent with the observation that G6PD-deficient mice are not more susceptible to infection than animals with normal enzyme activity [34]. It has been postulated that in cells other than erythrocytes, the intra‒mitochondrial NADPH generation may be sufficient to compensate the lack of its production in the pentose phosphate pathway making G6PD deficiency in these cells of poor clinical significance [35].

More interestingly, subgroups analysis showed that the prevalence of G6PD deficiency and G6PD normal activity was similar in patients with a past *H*. *pylori* infection indicating a similar behaviour for clearing infection.

Several limitations in our study should be taken into consideration. First, the patients' cohort investigated might pose some problems of sample representativeness as they were referred to a GI section for dyspeptic symptoms. However, the genetic and sociocultural homogeneity of the study population is great enough to minimize the effect of residual confounders. In addition, the chance to have gastric histological samples gave us the opportunity to accurately classify the type of *H*. *pylori* associated gastritis.

## Conclusion

Our results demonstrate for the first time that G6PD deficiency does not enhance patients’ susceptibility to acquire *H*. *pylori* infection and does not affect its eradication in Sardinia.
